# Validity of self-reported educational attainment collected as part of a telephone interview in a Norwegian dietary study

**DOI:** 10.1177/22799036261481566

**Published:** 2026-08-24

**Authors:** Jannicke Borch Myhre, Torunn Holm Totland, Lene Frost Andersen

**Affiliations:** 1 Department of Nutrition, 6305 University of Oslo, Oslo, Norway; 2 Department of Physical Health and Ageing, 25563 Norwegian Institute of Public Health, Oslo, Norway

**Keywords:** validity, self-report, education, educational level, educational attainment, Norway, interview

## Abstract

**Background:**

Self-reported educational attainment is a much-used variable in research, but few studies have addressed the validity of this information. This study assesses the criterion validity of self-reported educational attainment from a telephone interview by comparing it with registry-based data from Statistics Norway.

**Design and methods:**

Self-reported educational attainment was collected from 348 persons aged 18-80 years residing in Norway as part of a dietary study involving two 24-hour recalls conducted over the telephone during the spring of 2021. Information about officially registered educational attainment from Statistics Norway was later obtained for the same participants, and information from both sources was compared for 344 participants.

**Results:**

When dividing the participants into three educational groups, 77 % were classified in agreement with the officially registered educational attainment by self-report with regard to educational attainment. Twenty percent overreported their own educational attainment, while only 3% underreported. When dividing the participants into only two educational groups according to whether they had university/college education or not, 86% were in agreement with the officially registered educational attainment, 12% overreported their educational attainment and 2% underreported.

**Conclusions:**

The majority of participants were classified in agreement with the officially registered educational attainment by self-report, but overreporting was more common than underreporting. These results should be taken into consideration when using self-reported educational attainment in research.

## Introduction

Educational attainment is a much-used variable in research, both as a confounding variable and to make direct comparisons between groups with different levels of educational attainments.^1–3^ In many cases, educational attainment is self-reported,^4,5^ and as other self-reported data,^6,7^ it might be prone to misreporting. Despite its importance, the scientific literature has generally paid limited attention to the validity of self-reported data on educational attainment. However, the available existing studies suggest a significant degree of inconsistent reporting^8^ and misreporting, predominantly in the form of overreporting rather than underreporting.^9,10^ In the Norwegian context, the criterion validity of self-reported educational attainment has been evaluated in a population-based health study in the municipality of Tromsø in northern Norway.^11^ In data from the Tromsø study, self-reported educational attainment obtained from a questionnaire was compared to officially recorded data on education, finding overreporting of educational attainment by self-report. The question of the validity of self-reported educational attainment has also been addressed using data from the Norwegian Mother and Child Cohort Study, where the participating mothers’ self-reported educational attainment from a questionnaire was compared to official registries. The results showed a somewhat higher self-reported educational attainment compared to what was recorded in the national registries.^12^

The manner of administrating the questions about educational background might have implications for the validity of the response.^13^ Both the Tromsø study and the Norwegian Mother and Child Cohort Study used questionnaires for collecting self-reported data on educational attainment. In the present paper we evaluate the criterion validity of self-reported educational attainment collected as part of a telephone interview by comparing it to officially recorded educational attainment from a national registry, and study if misreporting varies according to sex, age and if the participants were born abroad.

## Method

### Sample and design

A sample of 800 persons aged 18 to 80 years was randomly selected from the Norwegian National Population Register and invited to participate in a dietary survey in the spring of 2021. The participants completed two telephone-administered 24-hour dietary recalls (24-HDR) and answered one online questionnaire about habitual consumption of a selection of food items. During the 24-HDRs the participants were asked to describe their intake of all foods and beverages the preceding day. After the first 24-HDR a question about self-reported educational attainment was included in the interview. The question was an open question asking for the participant’s highest completed education. The interviewer would then choose the appropriate educational category based on the participant’s answer.

Of the invited 800 persons, 21 were considered not qualified to participate (no address or telephone number, not alive anymore, moved out of the country, did not speak Norwegian or English) and 348 (45 %) participants completed two 24 HDR. None of the participants had missing information on self-reported educational attainment. The study was approved by the Norwegian center for research data (project no 370209) and by Statistics Norway. Verbal informed consent was obtained from all participants and formally recorded.

### Definition of educational attainment

The eight available levels for categorizing information about self-reported educational attainment collected during the dietary interviews are shown in Table 1. The original categories were later regrouped to three groups corresponding to the groups used by Statistics Norway (Table 1). High school and vocational school were grouped together as both were considered as corresponding to high school by Statistics Norway up to the year 2016. From 2016 and onwards, vocational school has been treated as a separate educational attainment in national statistics. When dividing the participants into two groups according to whether or not they had university/college education, everyone that reported/were registered with university/college education was included in the university/college group (Table 1). This group corresponds to “high education” (levels 5-8) in the International Standard Classification of Education from 2011 (ISCED 2011).^14^

**Table 1.** Classification of education by self-report and by Statistics Norway.

Original classification of educational level in the dietary study	Corresponding educational categories used by statistics Norway	Corresponding categories when dividing educational level into two groups
No education	Elementary or junior high school	No university/college
Less than 7 years of education		
Junior high school		
1-2 years of high school	High school/vocational school	
Completed high school		
Vocational school		
University/college education short (4 years or less)	University/college education	University/college
University/college education long (more than 4 years)		

When grouping the participants into three levels of educational attainment, participants not reporting/being registered with any education above junior high school were grouped in the category “elementary or junior high school” (corresponding to “low education” (levels 0-2) in ISCED 2011). Those that reported/were registered with at least one year of high school but not having any university/college education were included in the category “high school/vocational school” (corresponding to “medium education” (levels 3-4) in ISCED 2011). Finally, participants that reported/were registered with university/college education were included in the university/college group (corresponding to “high education”(levels 5-8) in ISCED 2011) (Table 1).

### Data on educational attainment from statistics Norway

In the present study, the underlying construct of interest was formal educational attainment. Registry-based data from Statistics Norway were used as the reference measure. Statistics Norway has a database encompassing the educational attainment of all individuals registered as residents in Norway as of October 1 the preceding year.^15^ Educational data are collected from various sources such as the county municipal admission system to upper secondary education, the Database for Statistics on Higher Education, the Norwegian Centre for Research Data, and the administrative systems of various higher education institutions. The database is also supplemented with data from the National Diploma Database, the Health Personnel Register, the Database of Foreign Nationals, the Norwegian Agency for Quality Assurance in Education and the Norwegian Labor and Welfare Administration.^15^

To obtain the registered educational attainment from Statistics Norway, a file including the participants’ national identity number, sex, age, information about if they were born in Norway or not (yes or no variable) and self-reported educational attainment was transferred from the University of Oslo to Statistics Norway. Statistics Norway then returned the file with information about the registered educational attainment of each participant. In this file, the national identity number was removed so that the data could no longer be linked to identifiable individuals or to the data material from the rest of the dietary study.

### Statistical analyses

Self-reported data were compared to the data registered by Statistics Norway using frequency tables and stratification into subgroups. Participants were regarded as “misclassified” if they were grouped in a different educational group by Statistics Norway than by self-report. The McNemar test was used for pairwise comparisons when educational attainment was dichotomized into two categories, whereas the McNemar–Bowker test was used for pairwise comparisons using three categories of educational attainment. The chi-square test was used to compare differences in agreement between self-reported and officially registered educational attainment according to sex, age, and immigration status. Results with p<0.05 were considered statistically significant.

## Results

Four persons had missing data on educational attainment in the file from Statistics Norway and were excluded from the calculations. Characteristics of the included participants are shown in Table 2.

**Table 2.** Background characteristics of study population.

Background variable	Dietary study (n=344)^1^	Statistics Norway (n=344)	p
Sex			
Male, n (%)	170 (49%)	​	​
Female, n (%)	174 (51%)	​	​
Age			
18-30 years, n (%)	69 (20%)	​	​
31-40 years, n (%)	64 (19%)	​	​
41-50 years, n (%)	61 (18%)	​	​
51-60 years, n (%)	61 (18%)	​	​
61-70 years, n (%)	63 (18%)	​	​
71-80 years, n (%)	26 (8%)	​	​
Mean age, years, (SD)	47 (17)	​	​
Immigration status			
Born in Norway, n (%)	293 (85%)	​	​
Not born in Norway, n (%)	51 (15%)	​	​
Educational level			
Junior high school or less, n (%)	7 (2%)	​	​
1-2 years of high school, n (%)	16 (5%)	​	​
Completed high school, n (%)	57 (17%)	​	​
Vocational school, n (%)	75 (22%)	​	​
University/college education short (≤4 years), n (%)	123 (36%)	​	​
University/college education long (> 4 years), n (%)	66 (19%)	​	​
Educational level (three categories)			<0.001^2^
Junior high school or less, n (%)	7 (2%)	38 (11%)	​
High school/vocational school, n (%)	148 (43%)	149 (43%)	​
University/college education, n (%)	189 (55%)	157 (46%)	​
Educational level (two categories)			<0.001^3^
No university/college education, n (%)	155 (45%)	187 (54%)	​
University/college education, n (%)	189 (55%)	157 (46%)	​

^1^Sex, age and immigration status were collected from the National Population Register, educational attainment was self-reported in the dietary interviews.

^2^Difference in educational level between Statistics Norway and self-report, McNemar-Bowker Test.

^3^Difference in educational level between Statistics Norway and self-report, McNemar Test.

### Categorization into three educational groups

When using all the three educational categories from Statistics Norway, a statistically significant difference was seen between the self-reported educational attainment and the officially registered educational attainment (*x*^2^= 44.49, df = 3, p < 0.001, Table 2). Seventy-seven percent of the participants reported having the same level of education as the information registered by Statistics Norway (Table 3). The reporting accuracy varied according to some of the studied subgroups of participants. No difference was seen between men and women (Table 3), but the percentage of participants classified in agreement with the officially registered educational attainment varied according to age group, ranging from 68% for participants 61-70 years of age to 90% for participants 41-50 years of age (p = 0.03). A higher degree of misclassification was seen for immigrants than for persons born in Norway (p = 0.05) (Table 3).

**Table 3.** Classification of participants into three or two educational categories based on self-reported education and official statistics (n=344).

Background variable	n	Three educational categories^1^		Two educational categories^2^	
		Classified in accordance with official statistics by self-report^3^, n (%)	Misclassified by self-report^3^, n (%)	Classified in accordance with official statistics by self-report^3^, n (%)	Misclassified by self-report^3^, n (%)
All	344	266 (77%)	78 (23%)	296 (86%)	48 (14%)
Sex					
Men	170	132 (78%)	38 (22%)	146 (85%)	24 (15%)
Women	174	134 (77%)	40 (23%)	150 (85%)	24 (15%)
p^4^	​	0.89		0.93	
Age group					
18-30 years	69	52 (75%)	17 (25%)	61 (88%)	8 (12%)
31-40 years	64	52 (81%)	12 (19%)	58 (91%)	6 (9%)
41-50 years	61	55 (90%)	6 (10%)	57 (93%)	4 (7%)
51-60 years	61	42 (69%)	19 (31%)	49 (80%)	12 (20%)
61-70 years	63	43 (68%)	20 (32%)	49 (78%)	14 (22%)
71-80 years	26	22 (85%)	4 (15%)	22 (85%)	4 (15%)
p^4^	​	0.03		0.09	
Immigration status	​	​	​	​	​
Born in Norway	293	232 (79%)	61 (21%)	256 (87%)	37 (13%)
Born abroad	51	34 (67%)	17 (33%)	40 (78%)	11 (22%)
p^4^	​	0.05		0.09	

^1^Three educational categories: “junior high school or less”, “high school/vocational school”, “university/college”.

^2^Two educational categories: “no university/college”, “university/college”.

^3^Self-reported educational attainment obtained from a telephone interview.

^4^Difference in proportion of participants classified in agreement with the officially registered educational attainment by self-report according to sex, age or immigration status., chi square.

**Figure 1.**
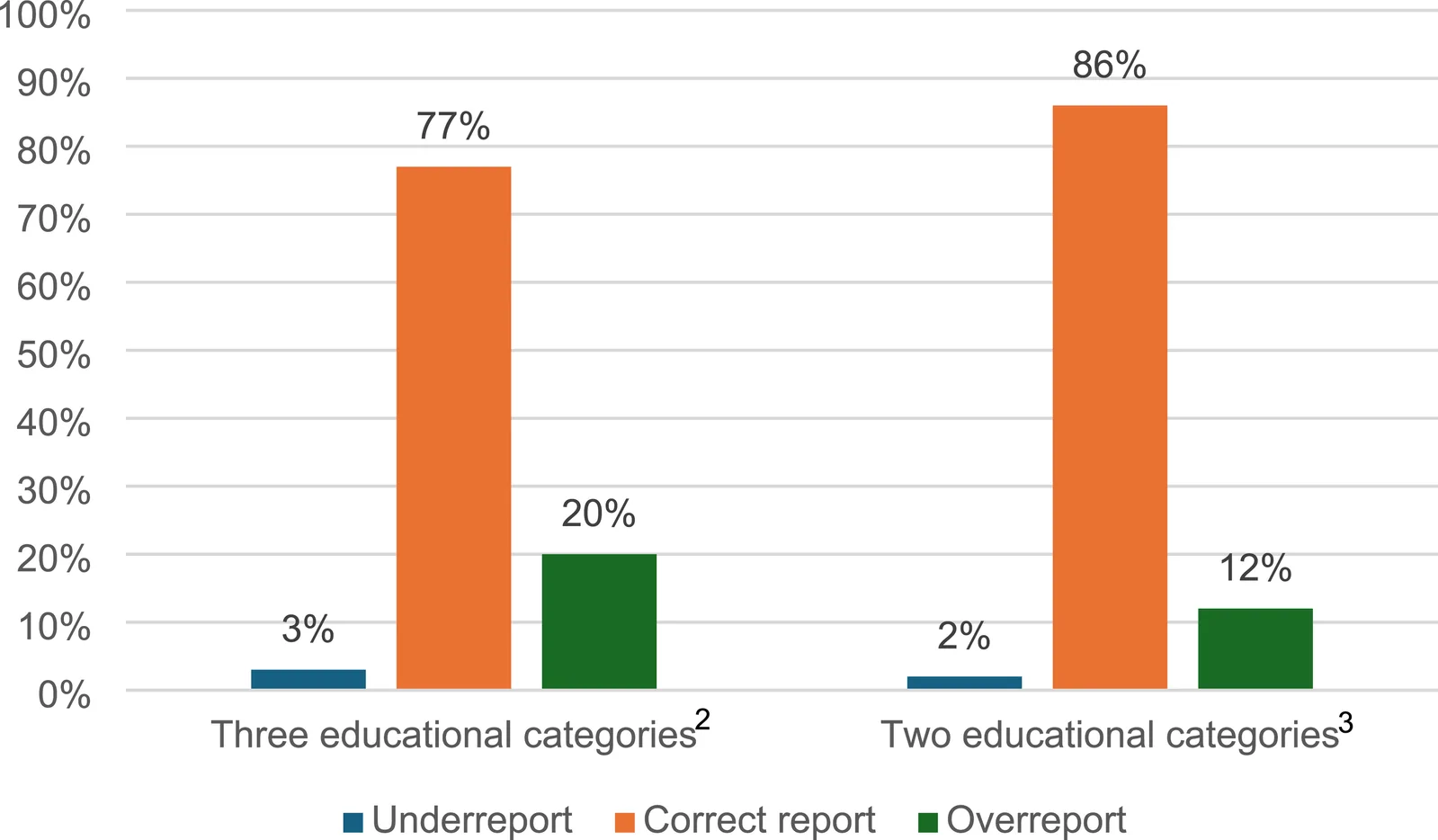
Criterion validity of self-reported^1^ compared to officially registered education, in two or three educational levels (n=344). 1. Self-reported educational attainment obtained from a telephone interview. 2. Three educational categories: “junior high school or less”, “high school/vocational school”, “university/college”. 3. Two educational categories: “no university/college”, “university/college”.

The results showed a clear tendency towards overreporting of educational attainment as 20% of the participants reported a higher educational attainment than what was officially registered by Statistics Norway while only 3% reported a lower than officially registered educational attainment (Figure 1).

The majority of participants (71%) who reported having “junior high school or less” were also registered with this level of education by Statistics Norway. Among those reporting “high school or vocational school” 76% were classified in agreement with the officially registered educational attainment, but 19% were registered as having “junior high school or less” by Statistics Norway. Finally, among those reporting a university or college education, 79% were also registered with this kind of education by Statistics Norway, leaving 21% registered by Statistics Norway as having “high school or vocational school” or “junior high school or less” (Table 4).

**Table 4.** Comparison of self-reported educational attainment and registered educational attainment by statistics Norway (n=344).

Self-reported^1^ educational attainment (n=344)	Educational attainment obtained from statistics Norway (n=344)	n (%)
Junior high school or less, n =7 (2%)	Junior high school or less	5 (71%)
	High school/vocational school	2 (29%)
	University/college	0
High school/vocational school, n=148 (43%)	Junior high school or less	28 (19%)
	High school/vocational school	112 (76%)
	University/college	8 (5%)
University/college, n=189 (55%)	Junior high school or less	5 (3%)
	High school/vocational school	35 (19%)
	University/college	149 (79%)

^1^Self-reported educational attainment obtained from a telephone interview.

### Categorization into two educational groups

Also when reducing the number of educational categories to two groups according to whether or not the participants had a university or college education, a statistically significant difference was seen between the self-reported educational attainment and the officially registered educational attainment (*x*^*2*^ = 21.33, df = 1, p < 0.001, Table 2). Eighty-six percent of the participants were classified in agreement with the officially registered educational attainment by self-report (Table 3).

Twelve percent overreported their own educational attainment while only 2% reported not having university or college education even though they were registered with this kind of education by Statistics Norway (Figure 1). Overall, 46% of the sample had university or college education according to the results from Statistics Norway (Table 2). This was nine percentage points lower than from the self-reported data on educational attainment from the telephone interviews (55%). Differences according to age and immigration status (both p = 0.09) were less prominent when participants were grouped into two educational groups.

## Discussion

This study compared self-reported educational attainment and registered educational attainment by Statistics Norway in a sample of 344 participants aged 18-80 years residing in Norway. When using all the available categories for educational attainment, the majority of participants (77%) were classified in agreement with the officially registered educational attainment by self-report. Still, a substantial proportion (23%) misreported their own educational attainment, and overreporting (20%) was more common than underreporting (3%).When dividing educational attainment into two groups (university/college education or not), the difference between registered and self-reported educational attainment became smaller, and 86% of the participants were classified in agreement with the officially registered educational attainment. No differences were seen between men and women, but some age groups were more prone to erroneous self-reporting than others.

Self-reported educational attainment is a frequently utilized variable in research, often serving as an indicator of socioeconomic status. Due to its widespread use, it is crucial to assess the validity of self-reported data on educational attainment. Studies from both the US and Great Britain have shown misreporting in the form of over-reporting rather than underreporting of educational attainment when using self-report.^9,10^

In the Tromsø study, conducted among individuals aged 40 years and older residing in the municipality of Tromsø in northern Norway, self-reported educational attainment obtained from a questionnaire was compared to recorded data on education from Statistics Norway, in a similar manner to the present study.^11^ Overreporting of educational attainment was also found in that study with 49% of the participants having university/college education by self-report, compared to 43% according to national statistics. These results are quite similar to the nine percentage points difference between self-reported and officially registered proportion of participants with a college/university education in the present study. In addition, misreporting in the Tromsø study was more prevalent among individuals aged 53-62 years. This is also in line with the findings in the present study that misreporting was most common in participants aged 51-70 years. In contrast to our findings, the Tromsø study observed a higher degree of misreporting among women than among men, whereas we found no sex difference. Others have found that men and women tend to be affected by different types of social desirability bias when participating in surveys. While men tend to be prone to more positively biased towards intellectual, social and emotional qualities, women are more prone to social desirability bias for qualities related to responsibility and interpersonal relationships.^16^ This would imply a higher tendency to overreport educational attainment in men than women, but was not seen in our data. Self-reported educational attainment was also evaluated in questionnaire data from The Norwegian Mother and Child Cohort Study (MoBa). The results showed that the participating mothers tended to overreport rather than underreport their own educational attainment. The difference largely consisted of mothers reporting more graduate tertiary education and less undergraduate tertiary education than in the registry. This distinction between different levels of college/university education was not made in the present study. Due to the relatively low number of participants, we did not separate the different age groups into men and women, but in accordance with data from the mothers in the MoBa study, the misclassification rate in the age groups including potentially childbearing mothers (age 18-40 years of age) was quite low, and lower than in the sample as a whole.

Although the basis of comparison from other similar studies is limited, the results from the present study seem to be in line with previous studies collecting self-reported data on educational attainment from questionnaires, suggesting that the tendency to overreport is present with both methodologies; questionnaire and interview.

### Reasons for misclassification

Even though most of the self-reported educational attainments were in accordance with the data registered by Statistics Norway, misreporting also occurred. The reasons for this are likely to be varied. Some participants might intentionally overreport their educational attainment to impress the interviewer/researcher. Others might misreport due to differences in definitions and interpretations. For instance, Statistics Norway only acknowledges completed courses, whereas the participants may consider their education nearly completed and therefore worthy of mentioning even though they skipped the last exams. Likewise, work-related continuing education courses might have been regarded as higher education by the participant, but not by Statistics Norway. Moreover, Statistics Norway redefined the definition of college or university education in 1998/1999,^15^ changing the requirement from one year of college or university to be enough to count as college or university education to two years. Consequently, a person who for instance completed one year of medical school after 1998/1999 would not be regarded as having a university or college education. In the present study it was not possible to evaluate the effect of this change. However, in a larger subsequent study including 1964 participants, the grouping of educational attainment was changed to accommodate the change in definition by Statistics Norway. Only a two percentage points difference was seen between the proportion of participants having a university/college education taking into account the change in definition after 1998/1999 and the estimates not taking this change into account (unpublished results). Hence, the change in definition is not likely to have influenced the results in the present study to a large extent. In addition, some educations have changed level over time as educational programs not previously regarded as being at the college/university level are now part of college/university programs. In an interview setting, proper training of interviewers is important to minimize the risk of misunderstandings and misinterpretation.

A higher proportion of misclassification was observed among immigrants, likely due to differences in educational systems and interpretation across countries. Language challenges might also have a played a role for some participants. In addition, documenting completed educations for correct classification in national statistics might be more challenging for individuals educated abroad.

### Consequences of misclassification

Awareness of misreporting of educational attainment is important in population studies, where the percentage of participants having completed university or college education is often compared to national statistics and used as an indicator of the generalizability of the results. However, overreporting by participants can lead researchers to perceive the results as less representative than they actually are. In the present study, 55% of the participants self-reported a college and university education compared to 46% based on nationally registered data by Statistics Norway. The age adjusted proportion of adults having a college or university education in Norway at the time of data collection was 37%. Hence, although the sample still consisted of a higher proportion of persons with higher education than the national average, the difference was smaller when using educational data from Statistics Norway.

Also in studies investigating differences in various outcomes according to educational attainment or using educational attainment as a covariate, correct categorization of participants is important, as incorrect classification will likely attenuate findings.^11^

When collecting self-reported educational data, it is important to know what groups of the population that may be more prone to misreporting than others. In the present study, immigrants and individuals aged 51-70 years seemed somewhat more likely to misreport than others. Hence, extra attention to correct classification of educational attainment may be in place when collecting information from these groups. This approach is likely to be easier to take into consideration when performing interviews than when using self-administered questionnaires.

### Strengths and limitations

The strengths of this study include the nationally representative sample of both men and women across a large age span and the availability of registered educational attainment by Statistics Norway. Most published studies assessing the validity of self-reported educational attainment have evaluated information collected from questionnaires.^11,12^ In this study, we expand the knowledge base to also include information collected through telephone interviews.

The study also has some limitations. Even though the sample started out as representative of the Norwegian population, the participation rate of 45% shows that a large proportion of the sample did not participate. The investigated sample included a lower percentage of persons without higher education than the general Norwegian population. As the agreement between reported and registered educational attainment seemed somewhat higher in participants with college/university education, a more representative sample of the population would have been likely to result in lower agreement than what we observed.

It would also have been interesting to study differences in various study outcomes, such as differences in intake of food groups or nutrients, using self-reported and officially registered educational attainment. This was, however, not possible as the data on officially registered educational attainment was only linked to sex, age and whether the participant was born in Norway or not in addition to the self-reported educational attainment. Hence, we could not make such comparisons.

## Conclusion

The present validation of self-reported educational attainment collected from telephone interviews compared to officially registered educational attainment, showed that most participants in the study reported an educational attainment in accordance with registered official information. Still, a substantial proportion of participants misreported their own educational attainment, and there was a clear tendency towards overreporting rather than underreporting. This is similar to what has been found in other studies using self-reported information about educational attainment from questionnaires. The findings might have implications both for studies using self-reported educational attainment as an indicator of the representativeness of the study sample and for studies investigating differences in various health outcomes according to educational attainment or using educational attainment as a covariate.

## Data Availability

The datasets used and/or analyzed during the current study are available from the corresponding author on reasonable request.*
